# Reduced Axonal Transport and Increased Excitotoxic Retinal Ganglion Cell Degeneration in Mice Transgenic for Human Mutant P301S Tau

**DOI:** 10.1371/journal.pone.0034724

**Published:** 2012-04-04

**Authors:** Natalie D. Bull, Alessandra Guidi, Michel Goedert, Keith R. Martin, Maria Grazia Spillantini

**Affiliations:** 1 Cambridge Centre for Brain Repair, Department of Clinical Neurosciences, University of Cambridge, Cambridge, Cambridgeshire, United Kingdom; 2 Department of Ophthalmology, University of Cambridge, Cambridge, Cambridgeshire, United Kingdom; 3 Cambridge National Institute for Health Research Biomedical Research Centre, University of Cambridge, Cambridge, Cambridgeshire, United Kingdom; 4 Medical Research Council Laboratory of Molecular Biology, Cambridge, Cambridgeshire, United Kingdom; The University of Sydney, Australia

## Abstract

The effects of tau hyperphosphorylation and aggregation on axonal transport were investigated in the optic nerve of mice transgenic for human mutant P301S tau. Transport was examined using cholera toxin B tracing. Retrograde transport was reduced in transgenic mice at 3 and 5 months of age, when compared to C57/Bl6 control mice. Anterograde axonal transport was also reduced in 3-month-old transgenic mice. Mild excitotoxic injury of retinal ganglion cells resulted in greater nerve cell loss in retinas from 3- and 5-month old P301S transgenic mice, when compared to controls. In conjunction with the detection of abnormal tau in the optic nerve in human and experimental glaucoma, the present findings suggest that tau hyperphosphorylation and aggregation may constitute targets for neuroprotective therapies in glaucoma as well as tauopathies.

## Introduction

Microtubule-associated protein tau is believed to play a role in the assembly and stabilisation of microtubules [1]. Its binding to microtubules is negatively regulated by phosphorylation. In several neurodegenerative diseases, tau protein assembles into abnormal filaments, where it is abnormally hyperphosphorylated. The identification of *MAPT* mutations in inherited frontotemporal dementia and parkinsonism linked to chromosome 17 (FTDP-17T) has established that dysfunction of tau protein is sufficient to cause neurodegeneration and dementia [2]–[4]. Most mutations are located in or near the repeat region of tau, which is essential for microtubule binding. Consequently, mutant tau has been shown to exhibit reduced microtubule binding [5], [6], suggesting that a partial loss of function may be required for filament assembly and abnormal hyperphosphorylation. In addition, many *MAPT* missense mutations also increase the propensity of tau protein to assemble into filaments [7], [8].

Transgenic models expressing human mutant tau in nerve cells have been useful for investigating the toxicity of tau aggregation and how dysfunctional tau interferes with axonal transport. Defective axonal transport, both anterograde and retrograde, has been described in some of these models [9], [10]. We previously showed that in retinal ganglion cells from a mouse line transgenic for human mutant P301S tau, dynactin, which is required for the binding of cargo to dynein motor proteins, is abnormally distributed [11]. We also showed that recombinant human tau promotes attachment of the dynactin complex to axonal microtubules, indicating a potential role for tau in axonal transport. Dynein-mediated retrograde axonal transport of target-derived neurotrophic factors, such as brain-derived neurotrophic factor (BDNF), is essential for the survival of adult retinal ganglion cells (RGCs) [12]. Disruption of BDNF transport contributes to retinal ganglion cell death in glaucoma [13], [14] where, in an experimental model, we have previously described an altered distribution of dynein [15].

Here we examined the effects of human mutant P301S tau expression in retinal ganglion cells on axonal transport and nerve cell survival *in vivo*. In this transgenic line, human mutant P301S tau is expressed downstream of the murine Thy1 promoter [16]. By 5 months of age, homozygous mice exhibit widespread tau aggregation and neurodegeneration, accompanied by behavioural impairment and motor dysfunction. We previously described the presence of aggregated human mutant tau in RGCs of these mice [17]. However, nerve cell loss was not observed and retrograde axonal transport in retinal explant cultures appeared to be normal. We now show that aggregation of human mutant P301S tau in RGCs is associated with a reduction of both anterograde and retrograde axonal transport *in vivo*, and with a markedly increased effect of mild excitotoxic injury.

## Materials and Methods

### Ethics statement

Animals had unrestricted access to food and water, and were maintained on a 12 h light/dark cycle. All experiments were carried out in accordance with the UK Home Office Regulations for the Care and Use of Laboratory Animals and the UK Animals (Scientific Procedures) Act 1986. All methods were approved by the University of Cambridge Animal Ethics Committee (project licences 80/1914 and 80/2360).

### Animals

Homozygous male mice transgenic for human mutant P301S tau were used at 1, 3 and 5 months of age. At 5 months, these mice exhibit abnormal motor behaviour. No such abnormality is present in animals aged 3 months or 1 month. Filamentous inclusions made of hyperphosphorylated tau are present in the central nervous system of 3- and 5-month old transgenic mice [18]. We previously described the expression of human mutant P301S tau in RGCs and the formation of tau inclusions [17]. Based on staining with the fluorescent Congo red derivative FSB [19], the first filamentous tau aggregates in RGCs were observed in 7-week old mice. Age- and sex-matched C57/Bl6 mice (Harlan, UK) were used as controls. Animals had unrestricted access to food and water, and were maintained on a 12 h light/dark cycle. All experiments were carried out in accordance with the UK Home Office Regulations for the Care and Use of Laboratory Animals and the UK Animals (Scientific Procedures) Act 1986. They were approved by the University of Cambridge Animal Ethics Committee (project licence 80/1914).

### Tracing of anterograde axonal transport

Mice aged 1 month (P301S, n = 6; C57/Bl6, n = 6), 3 months (P301S, n = 5; C57/Bl6, n = 5) and 5 months (P301S, n = 6; C57/Bl6, n = 4) were used. The vitreous body of the left eye was injected with 2 µl of a 0.1% solution of cholera toxin B protein (CTB) conjugated to Alexa Fluor-555 in sterile phosphate-buffered saline (PBS) (Invitrogen Inc., Paisley, UK). Following the injection, the needle was held in place for 30 s, to prevent leakage. Mice were perfused intracardially 1 day after the injection.

### Tracing of retrograde axonal transport

Mice aged 3 months (P301S, n = 6; C57/Bl6, n = 6) and 5 months (P301S, n = 10; C57/Bl6, n = 4) were used. They were fixed in a stereotaxic frame (World Precision Instruments Ltd., Stevenage, UK), an incision was made through the scalp to expose the skull and Bregma identified as the zero point. One µl of a 1% solution of CTB conjugated to Alexa Fluor-555 was injected bilaterally into the superior colliculus (coordinates: anterior-posterior −2.92 mm and medio-lateral ±0.5 mm from Bregma; dorso-ventral −2 mm from the surface of the skull). Injections were performed over 2 min and the needle left in place for 1 additional min. Mice were perfused intracardially 3 days after the injection.

### Excitotoxic lesioning in vivo

Mice aged 1 month (P301S, n = 4; C57/Bl6, n = 4), 3 months (P301S, n = 6; C57/Bl6, n = 4), and 5 months (P301S, n = 7; C57/Bl6 n = 6) were used. One µl of 2 mM NMDA (N-methyl-D-aspartate; Sigma-Aldrich UK, Gillingham, UK) and 5 mM glycine (Sigma-Aldrich UK) in PBS was injected into the vitreous body of the left eye. The needle was held in place for 30 s following injection, to prevent leakage. This resulted in a concentration of 2 nmol NMDA and 5 nmol glycine per eye, which caused a mild excitotoxic injury of the retina [20]. Mice were perfused intracardially after 7 days.

### Quantification of retinal ganglion cells

**Figure 1:**
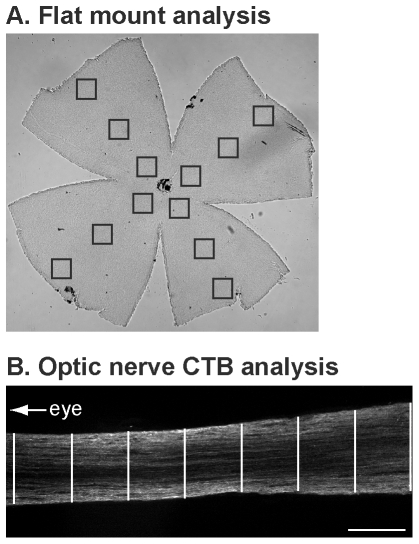
Quantification Methodology. (A), Retinal ganglion cell (RGC) survival following excitotoxic injury *in vivo* was quantified using retinal flat mounts immunohistochemically labelled for NeuN. Twelve images (3 per quadrant at central, medial and peripheral locations; approximate positions defined by boxes) were captured per retina using a 40× objective; NeuN-positive nuclei were counted in each image and their average number calculated for each retina. RGC loss was calculated compared to NeuN counts from the uninjured contralateral eye. (B), Axonal transport of fluorescent cholera toxin B (CTB) in the optic nerve was quantified by measuring average fluorescence intensity across the width of the optic nerve at 100 µm intervals along the full length of each nerve. A representative image is shown, with the white lines indicating example regions where average fluorescence intensity was measured. Scale bar, 100 µm.

The retinas of perfused mice were dissected and four radial cuts made in each retina, to facilitate whole mount staining and analysis. This was followed by a 2 h post-fixation in 4% paraformaldehyde/0.1 M PBS. The retinas were washed in PBS, followed by a 60 min incubation in blocking solution consisting of 0.2% Triton-X100 (Sigma-Aldrich UK) and 5% goat serum (Invitrogen Inc.) in PBS. They were then incubated for 24 h at 4°C with monoclonal anti-NeuN antibody (1∶500, Millipore, Watford, UK) in blocking solution, followed by washing in PBS. The retinas were incubated for 18 h at 4°C with Alexa Fluor-555 conjugated goat anti-mouse antibody (1∶1,000, Invitrogen Inc.) in blocking buffer. Following washing in PBS, the tissues were mounted on glass slides. Analyses were carried out blind with respect to treatment groups. The RGC layer was viewed under epifluorescent illumination and three images (central, medial and peripheral) per retinal quadrant captured using a 40× objective (Figure 1A). Each image sampled an area of 0.093 mm^2^; thus, with 12 images per retina (1.116 mm^2^), and given an explant area of 15.76±0.31 mm^2^, approximately 7% of the flat mount area of the retina was sampled. NeuN-positive cells were counted and the number of RGCs determined. The percentage loss of RGCs following excitotoxin administration was calculated by comparing the treated and untreated eyes.

### Excitotoxic lesioning in vitro

Retinas from mice aged 5 months (P301S, n = 4 mice; C57/Bl6, n = 4 mice; both retinas from each mouse were pooled) were dissociated into single cell suspensions using a papain dissociation system (Worthington Corporation, Lakewood, NJ, USA), following the manufacturer's instructions. The cells were plated at a density of 4×10^5^ cells/well (24-well plates) on poly-L-lysine (100 µg/ml; Sigma-Aldrich UK) and laminin (1 µg/ml; Sigma-Aldrich UK) coated glass coverslips in control medium (to make 25 ml: 24.2 ml Neurobasal-A medium, 500 µl B27 supplement, 62.5 µl L-glutamine, 125 µl gentamicin; all from Invitrogen Inc.) or in pH-balanced medium containing 100 µM glutamic acid (Sigma-Aldrich UK). After 3 days, the cells were fixed with 4% paraformaldehyde/0.1 M PBS and labelled with an anti-βIII tubulin antibody (1∶2000, Promega, Southampton, UK). The number of labelled cells in 10 fields of view per coverslip (4 replicate coverslips per N), using a 20× objective, was counted, and averaged over 4 coverslips for each sample. Cells were counted and the percentage of surviving cells determined by comparing cell counts between glutamate-treated and untreated control groups. An unpaired Student's t-test was used to compare retinal ganglion cell survival between P301S and C57/Bl6 cultures; a p value of less than 0.05 was considered significant.

### CTB binding to GM1 ganglioside receptor in retina and brain tissue

Sections of fixed retina (14 µm) and superior colliculus (30 µm) were obtained from 3 month old P301S and C57/Bl6 mice. Retinal sections were immunohistochemically labelled with a mouse monoclonal anti-βIII tubulin antibody (1∶2000; Promega UK, Southampton, UK), and brain sections immunohistochemically labelled with a mouse monoclonal anti-vGluT2 antibody (Abcam Plc, Cambridge, UK). An Alexa Fluor-488 conjugated goat anti-mouse secondary antibody (1∶1000, Invitrogen Inc.) was used to detect the primary antibody, and sections were exposed to Alexa Fluor-555 conjugated CTB protein (5 mg/ml; Invitrogen Inc) to visualise GM1 receptor binding. The retinal sections were imaged using laser scanning confocal microscopy (TCS-SPE, Leica Inc., Wetzlar, Germany), and brain sections were imaged using epifluorescent microscopy (DM6000 microscope; Leica Inc.).

### Quantification of the axonal transport of cholera toxin B

Optic nerves of perfused mice were dissected between the posterior globe and the optic chiasm, post-fixed overnight at 4°C, washed in PBS and cryo-preserved by overnight immersion in 30% sucrose at 4°C. Following embedding in OCT, 14 µm-thick longitudinal sections were cut using a cryostat and mounted onto microscope slides. They were immunohistochemically labelled for βIII tubulin (1∶2000, Promega), immunohistochemical visualisation of CTB was not required as the fluorophore was directly conjugated to the CTB protein. Analyses were carried out blind with respect to treatment groups. Optic nerve sections were visualised under epifluorescent illumination on a single Leica DM6000 microscope using a 10× objective and contiguous images along the length of each nerve captured using LAS AF software (Leica Inc.) and identical camera settings. Mean fluorescence intensity was measured across the width of each nerve at 100 µm intervals along its length (Figure 1B) and plotted as a line graph against distance (along the nerve), and the area under the curve (AUC) calculated using Prism software (GraphPad, La Jolla, USA). The AUC values were averaged across each experimental group and an unpaired Student's t-test used for statistical comparison of CTB transport; p<0.05 was considered significant. Fluorescence intensity was also measured along the length of a single optic nerve from an untreated right eye in each anterograde transport experiment to confirm background fluorescence was well below that detected in CTB-treated optic nerves.

### Tau immunohistochemistry

Optic nerve sections were immunohistochemically double-labelled with a rabbit polyclonal anti-βIII tubulin antibody (1∶1000; Covance, Harrogate, UK), and either mouse monoclonal antibody HT7 (1∶500; Pierce Protein Research Products, Thermo Fisher Scientific, Cramlington, UK) or phosphorylation-dependent mouse monoclonal anti-tau antibody AT8 (1∶1000; Source Bioscience Autogen, Calne, UK). The secondary antibodies were Alexa Fluor-647 conjugated goat anti-rabbit antibody (1∶1000, Invitrogen Inc.) and Alexa Fluor-488 conjugated goat anti-mouse antibody (1∶1000, Invitrogen Inc.). The sections were imaged using laser scanning confocal microscopy (TCS-SPE, Leica Inc.).

## Results

### Expression of hyperphosphorylated human mutant P301S tau in optic nerve

**Figure 2:**
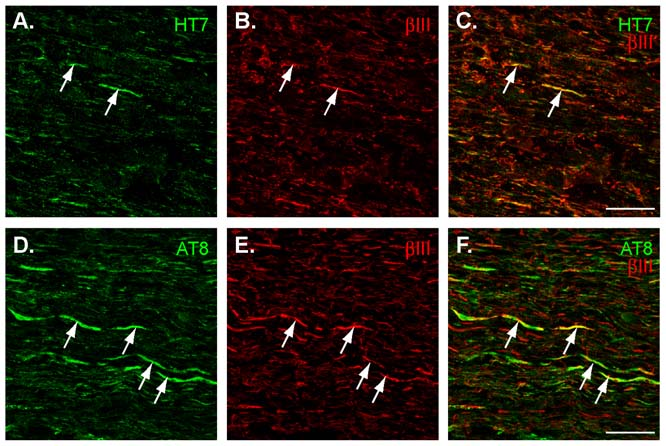
Expression of human tau in the optic nerve of mice transgenic for human mutant P301S tau. (A–C), Staining for human tau (A, green) and βIII tubulin (B, red) showed co-localisation in the axons of the optic nerve (C, overlay image of A and B; example from 1 month old mouse). (D–F), Staining for tau phosphorylated at S202/T205 (D, green) and βIII tubulin (E, red) showed co-localisation in the axons of the optic nerve (F, overlay image of D and E; example from 5 month old mouse). Arrows indicate examples of co-localisation. Scale bar, 20 µm.

We have shown previously that the P301S tau transgene is expressed by retinal ganglion cells (RGCs), under the Thy1 promotor, within the inner retina and nerve fibre layer [17]. Furthermore, it was demonstrated that expression of this transgene led to the accumulation and aggregation of hyperphosphorylated tau within RGC axons in the retina of these mice by the age of 2 months. In the current project, we extended these findings by probing the relevance of RGC axonal expression of mutant human tau in the optic nerve of P301S tau transgenic mice. We confirmed human tau was present in axons of retinal ganglion cells, as reflected by the co-labelling with antibodies HT7 (human tau specific antibody) and βIII tubulin (RGC marker; Figure 2A–C) in the optic nerve from at least 1 month of age. Double-labelling with anti-tau antibody AT8 and the anti-βIII-tubulin antibody established the presence of abundant hyperphosphorylated tau in RGC axons at both 3 and 5 months of age (Figure 2D–F), while the AT8 staining was lower at 1 month of age, consistent with our earlier study.

### Binding of cholera toxin B (CTB) protein to GM1 receptor in P301S tissue

**Figure 3:**
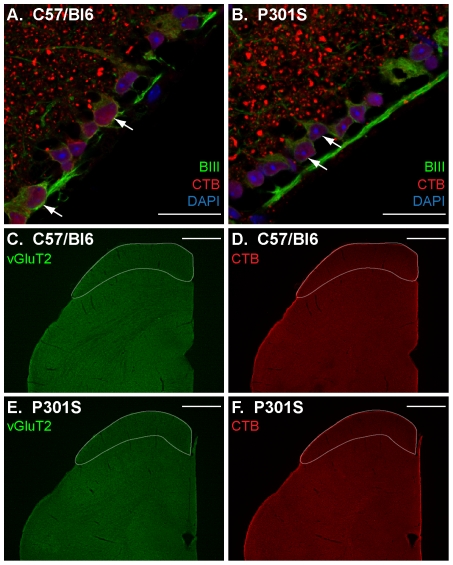
Binding of cholera toxin B (CTB) protein to GM1 receptor in P301S tissue. Fixed retinal (A–B) and brain (C–F) tissue was exposed to fluorescently tagged CTB (red) in order to visualise GM1 receptor binding in both C57/Bl6 control tissue (A, C–D) and P301S tissue (B, E–F). CTB binding was observed in RGCs within the retina (A–B), counterstained for the marker βIII tubulin (green; arrows indicate co-localisation), and punctate staining within the inner retina was also seen. Nuclei were counterstained with DAPI (blue). No difference in the pattern of CTB binding to the GM1 receptor in the retina was found between control (A) and P301S (B) tissue. Furthermore, no difference in the pattern of CTB binding to the GM1 receptor in the superior colliculus (outlined) of the brain was observed between control (D) and P301S (F) tissue. The RGC axon terminals in the superior colliculus were counterstained for the marker vGluT2 (vesicular glutamate transporter 2; green) in both control (C) and P301S (E) tissue. Scale bar, 25 µm A–B, 500 µm C–F.

Anterograde and retrograde transport of cholera toxin B (CTB) protein was used as a tool to assess RGC axonal transport. Binding of CTB to its receptor, the GM1 ganglioside receptor, on RGCs in both the retina and brain was examined to determine whether differences in receptor expression were apparent between P301S transgenics and controls. No difference in CTB binding to RGC somas, counterstained for the marker βIII tubulin (Figure 3A, B), or RGC terminals, counterstained for vGluT2 expression (Figure 3C–F), was observed between P301S and control tissue.

### Reduction of anterograde axonal transport in optic nerve from human mutant P301S tau transgenic mice

**Figure 4:**
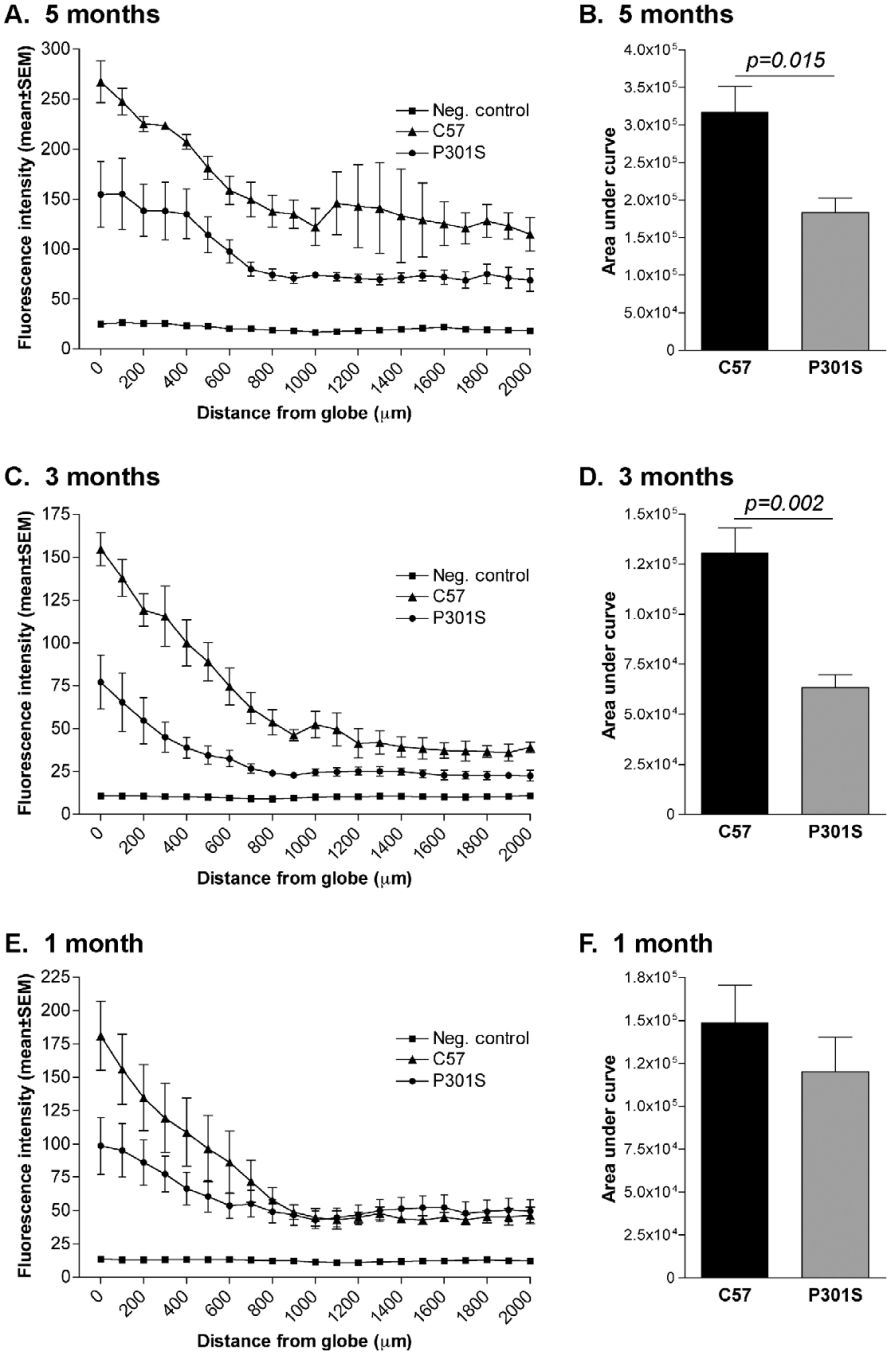
Anterograde axonal transport is reduced in optic nerve of mice transgenic for human mutant P301S tau mice. Fluorescent cholera toxin B was injected unilaterally into the vitreous and the amount transported in an anterograde direction measured in 5-month-old (A), 3-month-old (C) and 1-month-old (E) P301S tau transgenic and C57/Bl6 control mice. Fluorescence intensity appeared lower in transgenic mice at all ages, compared to controls. In optic nerves not exposed to cholera toxin B (negative control), only background fluorescence was measured (A,C,E). Statistical analysis of the area under the fluorescence intensity curve for each individual showed a significant reduction of anterograde axonal transport in optic nerves from P301S tau transgenic mice at 5 months (B) and at 3 months (D), but not at 1 month (F). Data are presented as mean±SEM.

Fluorescently tagged CTB was administered intravitreally and anterograde axonal transport in the optic nerve investigated 24 h later. Transgenic mice aged 5 months, 3 months and 1 month were used. Measurement of CTB fluorescence intensity showed that anterograde axonal transport was significantly impaired in transgenic animals aged 5 months (Figure 4A,B) and 3 months (Figure 4C,D), compared to controls. No significant impairment was detected in transgenic mice at 1 month of age (Figure 4E,F). The average fluorescence intensity along the untreated optic nerve showed that all experimental measurements were above background (Figure 4A,C,E).

### Reduction of retrograde axonal transport in optic nerve from human mutant P301S tau transgenic mice

**Figure 5:**
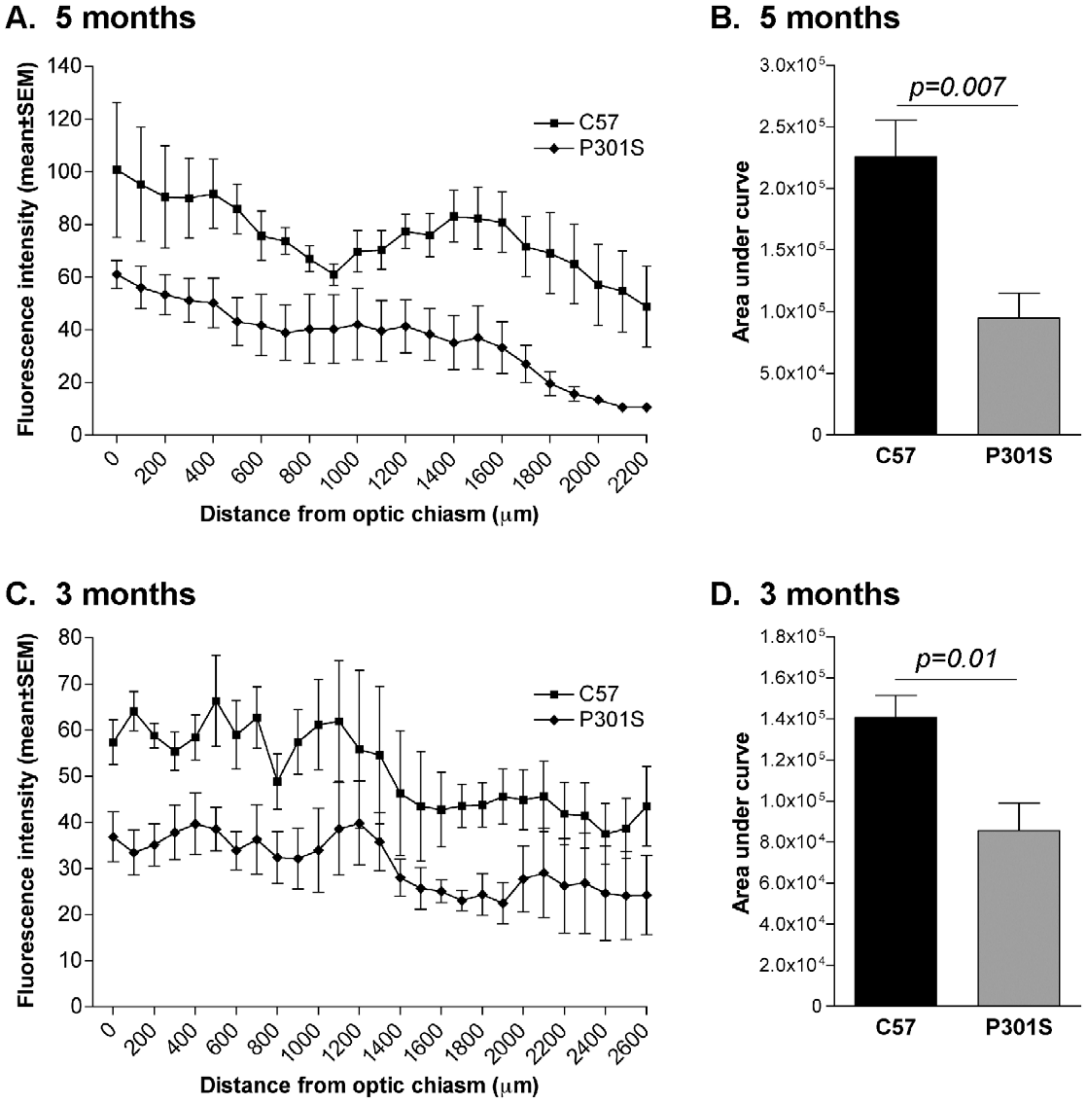
Retrograde axonal transport is reduced in optic nerve of mice transgenic for human mutant P301S tau. Fluorescent cholera toxin B was injected bilaterally into the superior colliculus and the amount transported measured in 5-month-old (A) and 3-month-old (C) P301S tau transgenic and C57/Bl6 control mice. Fluorescence intensity was lower along the length of the optic nerve in transgenic mice at all ages, compared to C57/Bl6 controls. Statistical analysis of the area under the fluorescence intensity curve for each individual showed a significant reduction in retrograde axonal transport in optic nerves from P301S tau transgenic mice at 5 months (B) and at 3 months (D), compared to controls. Data are presented as mean±SEM.

Fluorescently tagged CTB was injected into the superior colliculus and retrograde axonal transport in the optic nerve investigated 72 h later. Transgenic mice aged 5 months and 3 months were used; 1 month old animals were not used as their small size prevented accurate injection into the superior colliculus. Measurement of CTB fluorescence intensity showed that retrograde axonal transport was significantly impaired in transgenic mice aged 5 months (Figure 5A,B) and 3 months (Figure 5C,D), compared to controls.

### Increased susceptibility to excitotoxic injury of mice transgenic for human mutant P301S tau

**Figure 6:**
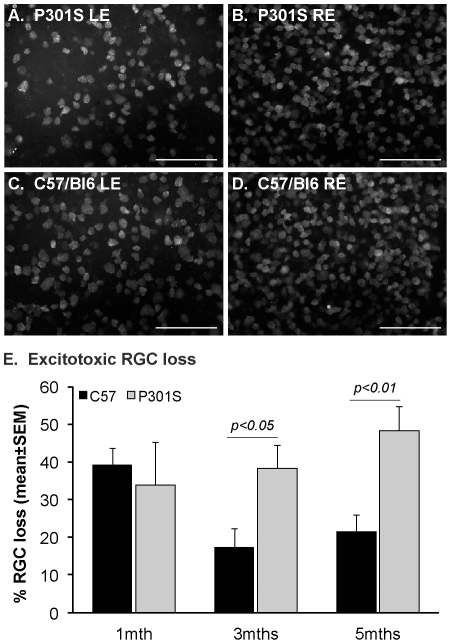
Reduced axonal transport in optic nerve of mice transgenic for human mutant P301S tau increases neuronal susceptibility to injury. Retinal ganglion cell (RGC) survival was quantified following a mild unilateral excitotoxic injury of the left eye (LE) by counting NeuN-positive nuclei in the RGC layer of the whole-mounted retina of 1, 3 and 5 month old mice (A–D). Percentage RGC loss was calculated by comparing the number of surviving RGCs in injured retinas to that of the uninjured right eye (RE). Representative images of NeuN-positive nuclei in injured P301S tau transgenic (A) and C57/Bl6 (C) retinas from 5 month old animals, compared to uninjured contralateral retinas (B and D), are shown as an example. Statistical analysis revealed a significant increase in RGC death following mild excitotoxic injury in P301S tau transgenic retinas, compared to C57/Bl6 control retinas (E), at both 3 months and 5 months of age; however no difference in RGC excitotoxic death was found at 1 month of age between control and transgenic retinas. N-methyl-D-aspartic acid (NMDA) was used as the excitotoxin. Scale bar, 100 µm.

To investigate the sensitivity of RGCs to injury, we exposed the retinas of wild-type and transgenic mice to a mild excitotoxic injury via intravitreal injection of 2 nmol NMDA plus 5 nmol glycine, as described [20]. One week after injection, the number of RGCs was reduced in the retinas of both human mutant P301S tau transgenic and C57/Bl6 control mice at 1, 3 and 5 months of age (Figure 6), however, while no difference in RGC loss was observed between 1 month old transgenic and control mice, significantly more RGCs died after mild excitotoxic injury in transgenic compared to control retinas at both 3 and 5 months of age (Figure 6E). In contrast, RGCs cultured from dissociated 5 month old P301S retinas, which disconnects them from their dependence upon retrograde neurotrophic supply, were no more susceptible to glutamate-induced death compared to RGCs isolated from C57/Bl6 retinas (percentage survival βIII tubulin^+^ cells in toxin-treated cultures compared to untreated cultures: P301S 76.22±4.83% compared to C57/Bl6 63.03±2.65%, mean±SEM, p>0.05, n = 4).

## Discussion

Hyperphosphorylation and aggregation of tau were associated *in vivo* with reduced axonal transport, both anterograde and retrograde, in the optic nerve of mice from a line transgenic for human mutant P301S tau. Axonal transport was assessed by measuring the distance travelled over time by fluorescently-tagged CTB. Impairment of axonal transport in transgenic P301S tau mice impacted negatively on the ability of RGCs to withstand a mild excitotoxic injury, resulting in enhanced nerve cell death. In comparison, when RGCs were dissociated and cultured *in vitro*, where cell survival was disassociated from reliance upon axonal transport, no difference in RGC survival was observed. In previous work in retinal explants of P301S tau transgenic mice [17], we failed to detect a reduction in retrograde axonal transport, possibly because this work was done in retinal explants where RGCs are axotomised.

In normal brain, the binding of tau to microtubules could regulate axonal transport. Tau has been reported to interfere with the binding of motor proteins to microtubules [21] and a tau gradient along the axon has been described, with the highest levels close to the synapse [22]. However, the effects of ablation of tau and increased tau expression on axonal transport have demonstrated that the rates of slow and fast transport along optic nerve axons *in vivo* are not significantly affected by the modulation of tau levels [23]. In squid axoplasm, where it was only weakly phosphorylated , monomeric tau did not affect axonal transport, even when present at 20-times its normal level [24]. Furthermore, the rates of axonal transport of human wild-type and mutant tau were similar in cultured cortical neurons [25]. It follows that the reduction of axonal transport described here was probably not due to the overexpression of monomeric human mutant tau, but resulted from tau hyperphosphorylation and aggregation. A similar conclusion was reached in studies of axonal transport in ventral roots of mice transgenic for tau with mutation R406W [9] and in dopaminergic substantia nigra neurons of mice transgenic for tau with mutation K369I [10]. Physiological phosphorylation of tau, as such, is probably not detrimental, since it occurs normally during foetal development [26] and hibernation [27]. However, the pathological hyperphosphorylation characteristic of human neurodegenerative diseases differs from that observed in foetal brain and during hibernation and may result in some toxic effects [1]. In support, overexpression of wild-type human tau in mouse neurons leads to some tau and impairment of axonal transport, in the absence of tau filament formation and significant neurodegeneration [28]–[30]. Aggregation of tau, in contrast, is likely to cause more extensive toxicity [1]. This is consistent with our finding that axonal transport was not impaired in 1 month old transgenic mice (Figure 4E, F), where less soluble tau was stained by the AT8 phosphorylation dependent anti-tau antibody in the RGC axons [17]. Impairment of axonal transport may result from the space-occupying nature of tau aggregates. In addition, more specific mechanisms may also be at work. We have previously shown that in the mouse line transgenic for human mutant P301S tau the dynactin complex is redistributed and exhibits a reduced interaction with microtubules [11]. It has also been reported that filamentous tau inhibits anterograde axonal transport by activating protein phosphatase 1 and glycogen synthase kinase-3, following increased exposure of amino acids 2–18 of tau, which comprise a phosphatase-activating domain [31], [32].

Reduced axonal transport may contribute to neurodegeneration by putting nerve cells under stress, limiting their ability to withstand injury. Thus, a mild excitotoxic insult, which kills fewer than 20% of RGCs in 3–5 month old control mice, killed 40–50% of RGCs in age-matched human P301S tau mice. Hyperexcitability of vulnerable nerve cells has been described as an early change along the path to neurodegeneration in many experimental animal models [33]. When survival was uncoupled from retrograde neurotrophic support by culturing dissociated RGCs *in vitro* (see Results for data values), or when axonal transport was not differentially affected (as in 1 month old animals; Figure 6E), excitotoxic injury equally affected the survival of wild-type and transgenic RGCs.

Impairment of axonal transport may be particularly deleterious for neurons such as RGCs, which rely heavily on retrograde trophic support. Interruption of retrograde axonal transport of target-derived BDNF and its receptor TrkB has been shown to contribute to the loss of RGCs in glaucoma [13], [14]. Pathological tau species have been detected in glaucoma [34], raising the possibility that dysfunction of tau may also be relevant in this disease.
